# Factors Associated With Overweight and Obesity Among Adolescents in South Asia: A Narrative Review of Recent Evidence

**DOI:** 10.1002/edm2.70372

**Published:** 2026-10-22

**Authors:** Tasnima Siddiquee, Bishwajit Bhowmik, Nayla Cristina Do Vale Moreira, Sanjida Binte Munir, Hafiza Nasrin, Tanjimul Islam, Fahmida Mahmud, Liya Quamrun Nesa, Shamima Akhtar, Shamim Khan, Farsana Rahman Khan, Mohammad Robed Amin, Abul Kalam Azad Khan

**Affiliations:** ^1^ Centre for Global Health Research, Diabetic Association of Bangladesh Dhaka Bangladesh; ^2^ University of Nord Bodo Norway

**Keywords:** adolescent, overweight, South Asia

## Abstract

**Introduction:**

Adolescent overweight and obesity are increasing across South Asia, contributing to diabetes and other noncommunicable diseases. Previous reviews have examined prevalence and selected associated factors, but evidence remains heterogeneous across countries, populations and adiposity definitions. This narrative review synthesised recent evidence on prevalence and factors associated with overweight and obesity among South Asian adolescents.

**Methods:**

We conducted a structured narrative review reported in accordance with the Scale for the Assessment of Narrative Review Articles. PubMed, Scopus and Google Scholar were searched for peer‐reviewed studies published from January 2015 to March 2025, supplemented by targeted searches through August 2026. Studies of adolescents aged 10–19 years and predominantly adolescent populations with overlapping age ranges were considered. Primary studies and evidence syntheses were analysed separately. Methodological quality was evaluated using design‐appropriate Joanna Briggs Institute critical appraisal checklists, and findings were synthesised narratively.

**Results:**

Thirteen publications were included: eight primary studies and five systematic reviews or meta‐analyses. Prevalence varied by population, setting, adiposity measure and classification system. Regional meta‐analytic estimates were 12.4% for overweight, 6.6% for obesity and 19.3% for combined overweight/obesity, although these were not directly comparable with adolescent‐specific primary studies. More consistent evidence concerned physical inactivity, sedentary behaviour and unhealthy dietary practices. Associations with socioeconomic position, urban residence, sex and gender, familial factors and media exposure were more limited and context dependent. Sleep, mental health, food insecurity and neighbourhood environments were insufficiently examined. Most primary evidence was cross‐sectional and school based.

**Conclusions:**

Adolescent overweight and obesity in South Asia are associated with behavioural, dietary and socio‐environmental factors within changing food and urban environments. Interpretation is limited by heterogeneous definitions, uneven country coverage and predominantly cross‐sectional designs. Standardised population‐based and longitudinal studies, including out‐of‐school adolescents, are needed to guide context‐specific prevention.

## Graphical abstract

Adolescent overweight and obesity are increasing across South Asia. Regional estimates indicate 12.4% overweight, 6.6% obesity and 19.3% combined overweight/obesity. Physical inactivity, sedentary behaviour and unhealthy diets show the most consistent associations, supporting context‐specific prevention and better longitudinal evidence.

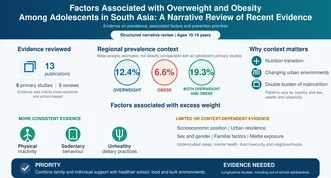

## 1 Introduction

The global prevalence of overweight and obesity among children and adolescents has risen markedly over the past four decades, with excess weight becoming an increasingly important public health challenge in low‐ and middle‐income countries (LMICs) [1]. Recent global evidence from 2033 studies conducted in 154 countries or regions estimated that 14.8% of children and adolescents had overweight, 8.5% had obesity and 22.2% had combined excess weight, although estimates varied substantially across countries, age groups and classification systems [2]. This epidemiological transition is particularly important in South Asia, a region of more than 1.9 billion people that continues to experience persistent undernutrition alongside a rapidly increasing burden of obesity and related noncommunicable diseases (NCDs) [3, 4]. A recent South Asian meta‐analysis estimated pooled prevalences of 12.4% for overweight, 6.6% for obesity and 19.3% for combined overweight/obesity among children and young people [5]. These estimates demonstrate a substantial regional burden but should not be directly compared with the global estimates because the underlying reviews differed in age ranges, study periods, sampling methods and adiposity classifications. Rapid urbanisation, economic development, nutrition transition and changing patterns of diet and physical activity are likely to be important contextual influences [6, 7].

Adolescence, defined by the World Health Organization as 10–19 years of age, is a critical developmental period characterised by rapid physiological and psychosocial changes, increasing autonomy and the establishment of health behaviours that may persist into adulthood [8]. Overweight and obesity during adolescence are associated with immediate physical and psychosocial consequences, including psychological distress, orthopaedic complications, dyslipidaemia, hypertension and insulin resistance [9]. Moreover, adolescent obesity is a strong predictor of obesity in adulthood and is associated with an increased subsequent risk of Type 2 diabetes, cardiovascular disease and certain cancers [10]. Preventing excess weight gain during adolescence may therefore reduce both the immediate metabolic consequences of obesity and the future burden of NCDs on health systems and economies.

The growing burden of adolescent overweight and obesity in South Asia must be considered within the region's double burden of malnutrition. Undernutrition, micronutrient deficiencies, overweight and obesity may coexist within the same country, community, household or individual [3, 4]. For example, an adolescent may have excess adiposity while remaining deficient in essential micronutrients. Early‐life undernutrition followed by rapid weight gain and exposure to energy‐dense, nutrient‐poor diets may also increase later metabolic vulnerability. Consequently, obesity‐prevention strategies in South Asia must promote healthy growth and nutritionally adequate diets without worsening persistent forms of undernutrition.

South Asia is also undergoing a rapid but uneven nutrition transition. Traditional dietary patterns are increasingly accompanied or displaced by ultra‐processed foods, fast foods, fried snacks and sugar‐sweetened beverages [6, 7, 11, 12]. These changes are influenced by food availability, affordability, urbanisation, motorised transport, digital‐media exposure and commercial marketing—not solely by individual choice [11, 12].

Although South Asian countries share some demographic and nutritional transitions, the region should not be treated as epidemiologically homogeneous. Afghanistan, Bangladesh, Bhutan, India, Maldives, Nepal, Pakistan and Sri Lanka differ considerably in economic development, urbanisation, food environments, cultural practices, gender norms, school participation and healthcare access. Available studies also vary in design, sampling frame, age range, sex distribution, rural–urban setting and representativeness. In addition, the WHO, International Obesity Task Force (IOTF), US Centers for Disease Control and Prevention (CDC), Indian Academy of Paediatrics and national BMI‐for‐age criteria may classify the same population differently [13, 14, 15]. BMI‐defined overweight and obesity are also conceptually distinct from central obesity measured using waist circumference or waist‐to‐height ratio. These methodological differences limit direct comparisons of prevalence and associated factors across studies and countries.

Previous systematic reviews and meta‐analyses have synthesised the prevalence and selected behavioural, dietary, socioeconomic and familial factors associated with childhood and adolescent overweight and obesity in South Asia [5, 6, 7, 16, 17]. However, many combined children and adolescents, focused on individual countries, or prioritised pooled prevalence over critical interpretation of the direction, quality and contextual applicability of reported associations. Review‐level findings may also overlap because the same primary studies can be included in multiple reviews. Comparatively limited attention has been given to differences according to sex, socioeconomic position, rural–urban residence, school participation, sampling frame, adiposity definition and methodological quality. Emerging influences—including sleep, psychosocial stress, academic pressure, food insecurity, neighbourhood environments, digital exposure and commercial food marketing—have not been consistently integrated into an adolescent‐focused regional synthesis.

An updated and critically interpreted synthesis is therefore needed to distinguish recurring regional patterns from context‐specific findings, evaluate the methodological limitations of the available evidence and identify populations and countries that remain underrepresented. Because most primary studies were cross‐sectional and could not establish temporality or causality, this review uses ‘associated factors’ rather than ‘determinants’ or ‘causal risk factors’, except when reproducing terminology used in cited publications.

This synthesis is intended to support public health researchers, clinicians, policymakers, and programme planners by distinguishing recurring regional patterns from country‐ and context‐specific evidence and identifying priorities for surveillance and adolescent obesity prevention across South Asia.

Accordingly, this narrative review aims to synthesise and critically evaluate recent evidence on the prevalence of and factors associated with overweight and obesity among adolescents in South Asia. Specifically, it seeks to: (1) describe variations in prevalence according to country, population, setting and adiposity classification; (2) synthesise evidence concerning physical activity, sedentary behaviour, dietary practices, sleep and psychosocial factors, familial and early‐life factors, sex and gender, socioeconomic position, food insecurity, rural–urban and built‐environment characteristics, and media and commercial influences; (3) examine regional, sex‐related, socioeconomic and rural–urban heterogeneity; and (4) identify methodological limitations and priorities for future research, policy and context‐specific public health interventions.

## 2 Methods

### 2.1 Review Design and Literature Search

This narrative review was conducted and reported in accordance with the Scale for the Assessment of Narrative Review Articles (SANRA) recommendations [18]. To enhance transparency, a structured literature search and critical narrative synthesis were undertaken to identify relevant evidence on the prevalence of and factors associated with overweight and obesity among adolescents in South Asia. A narrative‐review design was selected because the objective was to interpret heterogeneous evidence across behavioural, dietary, familial, biological and socio‐environmental domains rather than estimate a single pooled association. The included literature varied substantially in population, setting, adiposity definition, exposure measurement and analytical approach, making a critical narrative synthesis more appropriate than a formal meta‐analysis. Because this was not a systematic review, a protocol was not prospectively registered.

A structured search of PubMed, Scopus and Google Scholar was conducted for studies published between January 2015 and March 2025. Supplementary targeted searches were conducted through August 2026 to identify recently published South Asian evidence and literature addressing domains raised during peer review. These searches identified no additional eligible South Asian studies; therefore, the included‐study numbers and study‐selection flow remained unchanged. Recent global and non‐South Asian evidence identified during these searches was used only for contextual comparison in the Introduction and Discussion. The search strategy combined terms relating to:

- Overweight and obesity (‘overweight’, ‘obesity’, ‘adiposity’, ‘body mass index’, ‘BMI’, ‘BMI‐for‐age’, ‘central obesity’, ‘abdominal obesity’, ‘waist circumference’ and ‘waist‐to‐height ratio’).
- Adolescents (‘adolescent’, ‘teenager’, ‘youth’, ‘young people’ and ‘school‐going adolescent’).
- Associated factors (‘determinants’, ‘risk factors’, ‘predictors’, ‘associated factors’, ‘correlates’, ‘physical activity’, ‘physical inactivity’, ‘sedentary behaviour’, ‘screen time’, ‘diet’, ‘fast food’, ‘ultra‐processed food’, ‘sugar‐sweetened beverage’, ‘sleep’, ‘mental health’, ‘stress’, ‘academic pressure’, ‘food insecurity’, ‘socioeconomic status’, ‘family’, ‘parental’, ‘neighbourhood’ and ‘built environment’).
- Geographical setting (‘South Asia’, ‘India’, ‘Pakistan’, ‘Bangladesh’, ‘Nepal’, ‘Sri Lanka’, ‘Bhutan’, ‘Maldives’ and ‘Afghanistan’).

Controlled vocabulary, including relevant Medical Subject Headings, was used where applicable, together with free‐text terms. Search terms were adapted to the indexing and syntax of each database. Searches were limited to articles published in English. Reference lists of eligible articles and relevant systematic reviews were manually screened to identify additional publications. Recent global and non‐South Asian evidence was identified separately and used only to support contextual comparison in the Introduction and Discussion. The detailed database‐specific search strategies and supplementary search procedures are provided in Table S1.

### 2.2 Eligibility Criteria

Studies were eligible for inclusion if they:

- Reported prevalence estimates or factors associated with overweight or obesity.
- Included adolescents aged 10–19 years or a population explicitly described as adolescents in which the reported mean or median age fell within this range.
- Were conducted in one or more of the eight South Asian countries: Afghanistan, Bangladesh, Bhutan, India, Maldives, Nepal, Pakistan, and Sri Lanka.
- Used a recognised anthropometric measure, including BMI, BMI‐for‐age, waist circumference or waist‐to‐height ratio.
- Were published in a peer‐reviewed journal within the predefined review period.

Studies with broader or overlapping age ranges were eligible when adolescent‐specific findings were reported separately or could be clearly extracted. Given the limited regional evidence base, studies were also retained when the population was explicitly described by the authors as adolescents or predominantly comprised participants within the 10–19‐year age range, even if the complete age range was unavailable or included a small proportion of participants immediately outside this range. Such studies were clearly identified and interpreted cautiously. Their prevalence estimates were not considered directly comparable with estimates derived exclusively from participants aged 10–19 years.

Studies retained under these extended age‐eligibility provisions were clearly identified in the study‐characteristics table. Their age coverage, adiposity classifications, and limitations were considered when interpreting their findings and comparing prevalence estimates.

Both primary observational studies, including cross‐sectional, cohort, and case–control studies, and systematic reviews or meta‐analyses were considered. Primary studies constituted the principal evidence base for the synthesis of associated factors. Systematic reviews and meta‐analyses were analysed separately and used to establish the existing evidence base, compare regional prevalence estimates, identify additional relevant primary studies, and contextualise the findings. They were not combined with primary studies when assessing the consistency of associations, thereby reducing the risk of overlapping evidence and double‐counting.

BMI‐defined overweight and obesity were treated as the principal outcomes. Overweight, obesity and combined overweight/obesity were extracted separately wherever possible. Central obesity, defined using waist circumference or waist‐to‐height ratio, was considered a distinct secondary outcome and was not combined directly with BMI‐defined overweight or obesity.

Age‐ and sex‐specific BMI classifications, particularly the WHO 2007 Growth Reference, were preferred for adolescent populations. Studies applying alternative recognised classifications, including IOTF, CDC, national or adult BMI thresholds, were retained to reflect the heterogeneity of the regional evidence base. Studies using adult BMI thresholds were eligible when they involved older adolescents or populations explicitly described as adolescents; however, their prevalence estimates were interpreted cautiously and were not considered directly interchangeable with estimates based on BMI‐for‐age criteria. The classification system used by each study was recorded and considered when comparing findings.

Studies were excluded if they:

- Focused exclusively on children younger than 10 years or adults older than 19 years and did not include a predominantly adolescent population.
- Included mixed‐age populations without separately extractable adolescent findings and provided no evidence that the study population was predominantly adolescent or had a mean or median age within the 10–19‐year range.
- Lacked information on prevalence or associated factors.
- Were editorials, commentaries, conference abstracts, case reports or narrative opinion articles.
- Included fewer than 50 participants.
- Did not provide sufficient information to determine the population, outcome, or anthropometric measurement method.

### 2.3 Study Selection

Titles and abstracts of retrieved records were screened independently by two reviewers. Potentially relevant publications were assessed through full‐text review against the eligibility criteria. Disagreements were resolved through discussion and consensus, with the involvement of another member of the review team when necessary. Reasons for excluding articles at full‐text assessment were documented. Primary studies and review‐level publications were categorised separately throughout the selection process. The study‐selection process is summarised in Figure 1.

**FIGURE 1:**
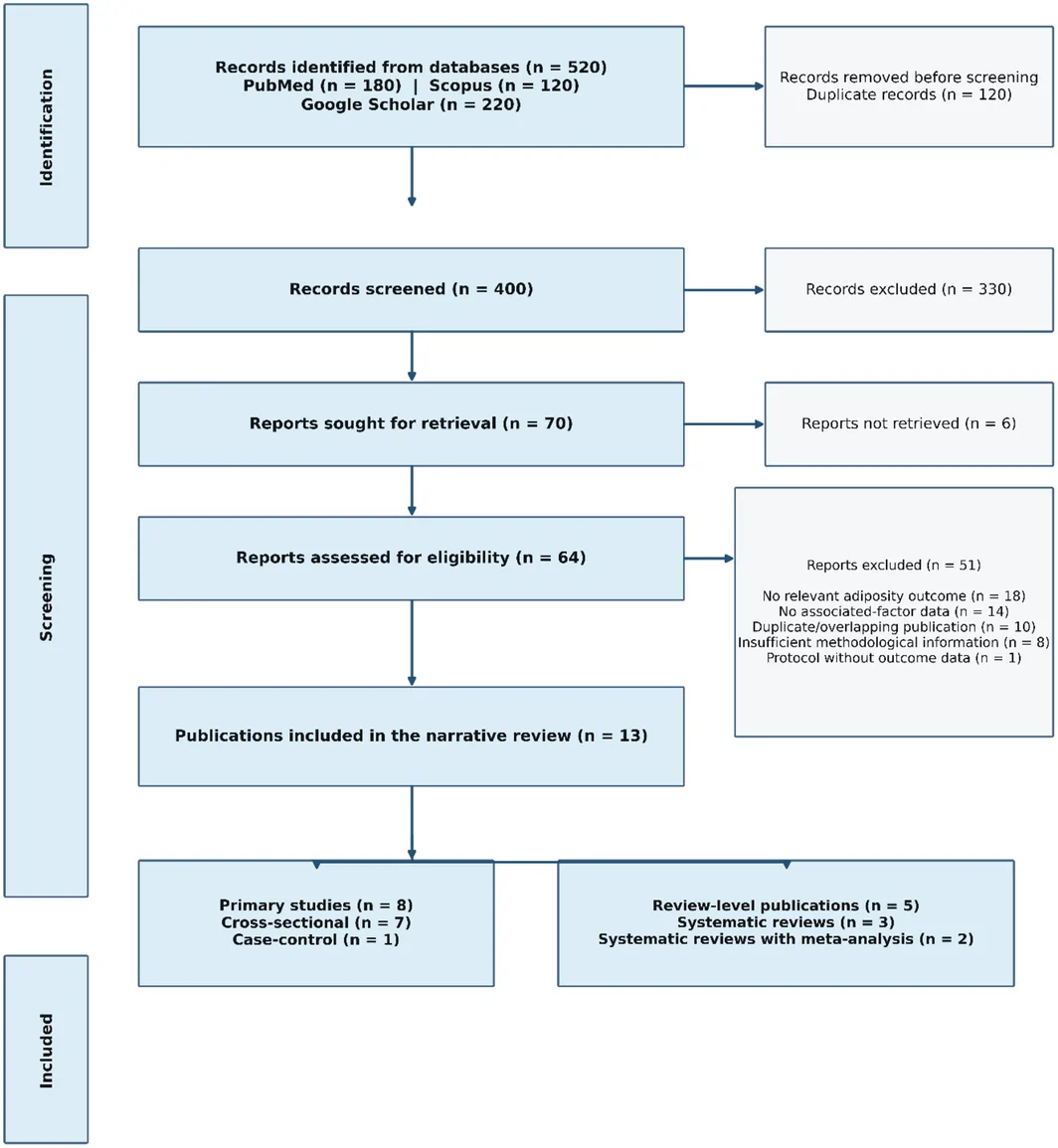
Study selection flow diagram.

### 2.4 Data Extraction

Data were extracted using a standardised data‐collection form. Information collected included:

- Author and publication year.
- Country and study period.
- Study design and setting.
- Sampling frame and representativeness.
- Sample size.
- Participant age range and sex distribution.
- Rural, peri‐urban or urban location.
- Measured or self‐reported anthropometry.
- Adiposity indicator and classification system.
- Prevalence of overweight, obesity, combined overweight/obesity or central obesity.
- Associated factor and its measurement.
- Crude and adjusted effect estimates.
- 95% confidence intervals.
- Covariates included in adjusted analyses.
- Methodological quality.

where studies reported results separately by sex, residential setting, socioeconomic position or adiposity outcome, these estimates were extracted separately. When information was unavailable or unclear, it was recorded as not reported rather than inferred. Data from systematic reviews and meta‐analyses were extracted into a separate evidence table and were not treated as independent primary observations.

### 2.5 Methodological Quality Assessment

To assess the robustness of the primary evidence, methodological quality was evaluated using design‐appropriate Joanna Briggs Institute critical appraisal checklists [19]. The JBI checklist for analytical cross‐sectional studies was applied to the seven cross‐sectional studies, and the JBI case–control checklist was applied to the case–control study (Tables S2A and S2B).

Quality assessment was conducted independently by two reviewers, and disagreements were resolved through discussion and consensus. Item‐level appraisal findings were recorded to identify potential limitations relating to participant selection, exposure and outcome measurement, identification and management of confounding, and appropriateness of statistical analysis.

Item‐level judgements were recorded as ‘yes’, ‘no’, ‘unclear’ or ‘not applicable’, as appropriate. No numerical quality scores or universal high‐, moderate‐ or low‐quality thresholds were applied because these are not prescribed by the JBI instruments. Instead, individual appraisal domains were considered when determining the interpretive weight given to each study.

Consistent with the narrative‐review approach, methodological quality was used to guide interpretation rather than as an automatic basis for study exclusion. Greater interpretive emphasis was placed on studies with representative samples, clearly defined outcomes, appropriate adjustment for confounding, and precise effect estimates. Findings from small, local, or methodologically limited studies were interpreted cautiously.

Systematic reviews and meta‐analyses were not formally appraised because they were included primarily as contextual and comparative evidence rather than as independent units contributing to the primary synthesis. Their scope, population, search period, and potential overlap with included primary studies were nevertheless considered when interpreting their findings.

### 2.6 Descriptive Analysis and Narrative Synthesis

Given the substantial heterogeneity in study designs, populations, settings, anthropometric measures, outcome definitions and analytical approaches, a narrative synthesis was undertaken [19]. Descriptive summaries were used to characterise study populations, prevalence estimates and reported associations.

Prevalence estimates were organised according to country, age, sex, setting, sampling frame, representativeness, anthropometric measure and classification system. Estimates of overweight, obesity, combined overweight/obesity and central obesity were presented separately and were not considered directly interchangeable.

Findings concerning associated factors were grouped into the following domains:

- Physical activity and sedentary behaviour.
- Dietary behaviours, nutrition transition and food environments.
- Sleep, mental health and psychosocial factors.
- Familial, parental, and early‐life factors.
- Sex‐ and gender‐related factors.
- Socioeconomic position and food insecurity.
- Rural–urban, neighbourhood, and built‐environment factors.
- Media exposure and commercial influences.

The original frequency‐based ‘vote‐counting’ approach, which calculated the percentage of studies reporting statistically significant associations, was discontinued because it did not account for sample size, effect magnitude, precision, confounder adjustment or methodological quality. Instead, the synthesis considered the direction and magnitude of associations, adjusted effect estimates, confidence intervals, sample size, representativeness, consistency across studies and methodological quality. Both statistically significant and non‐significant findings were considered where these were reported; however, failure to report a factor was not treated as evidence of a null association. For the study‐level evidence‐direction matrix, findings were classified as: (1) a positive association supported by a reported analysis; (2) an association suggested by the study authors but supported by insufficient quantitative evidence; (3) mixed findings or an unclear direction of association; or (4) not reported or apparently not assessed. The final category was not interpreted as evidence of no association because many studies did not report all exposures examined or all non‐significant results.

Studies were not assigned equal evidentiary weight. Greater emphasis was placed on larger, nationally representative or methodologically stronger studies, while results from small or highly selected samples were treated as context specific. Evidence was described as broadly consistent, mixed, limited or insufficient according to the direction and comparability of the available findings.

No quantitative meta‐analysis was undertaken because of substantial clinical and methodological heterogeneity. Accordingly, the findings should be interpreted as a critical narrative synthesis of the direction, context and quality of reported associations rather than as pooled causal estimates.

## 3 Results

### 3.1 Study Selection

A total of 520 records were identified through database searching, including PubMed (*n* = 180), Scopus (*n* = 120) and Google Scholar (*n* = 220). After removing duplicate records (*n* = 120) and records excluded for other reasons before screening (*n* = 30), 370 titles and abstracts were screened. Of these, 300 records were excluded, and 70 reports were sought for retrieval. Six reports could not be retrieved, leaving 64 full‐text reports for eligibility assessment.

Following full‐text assessment, 51 reports were excluded because they did not report relevant adiposity outcomes (*n* = 18), lacked information on associated factors (*n* = 14), represented duplicate or overlapping publications (*n* = 10), provided insufficient methodological information (*n* = 8), or were protocols without outcome data (*n* = 1). Ultimately, 13 publications were included in the narrative synthesis, comprising eight primary studies and five systematic reviews or meta‐analyses (Figure 1).

One primary study included participants aged 9–14 years [20]. Because most of its age range overlapped with the WHO‐defined adolescent period and it provided relevant evidence from an otherwise underrepresented urban population, it was retained as contextual mixed‐age evidence. Its findings were clearly identified and were not considered directly comparable with findings restricted to adolescents aged 10–19 years.

### 3.2 Characteristics of Included Studies

The eight primary studies comprised seven cross‐sectional studies and one case–control study. They included evidence from Nepal [12, 21], a multicountry study that included Bangladesh and Nepal [22], Sri Lanka [23], India [24, 25] and Bangladesh [20, 26] (Table 1). Sample sizes ranged from 60 to 51,597 participants, demonstrating substantial variation in statistical precision and representativeness.

**TABLE 1:** Characteristics and findings of primary studies examining factors associated with overweight and obesity among adolescents in South Asia.

Study	Country, setting and design	Participants	Adiposity measure and outcome	Prevalence	Principal associated factors and effect estimates	Interpretation
Gautam et al. (2024) [27]	Nepal; Nagarjun Municipality; school‐based cross‐sectional study	*n* = 768; Grades 8–10	Measured height and weight; BMI‐based overweight and obesity	Overweight: 5.6%; obesity: 0.8%; combined: 6.38% (95% CI 4.64–8.11)	Higher grade: AOR 3.61 (95% CI 1.26–10.30); no physical activity: AOR 4.97 (95% CI 2.38–10.34); no participation in school games or extracurricular activities: AOR 2.49 (95% CI 1.16–5.28); high‐calorie lunch was also associated	Local urban school sample; cross‐sectional design; several estimates had wide CIs
Sitaula (2023) [21]	Nepal; rural schools; cross‐sectional study	*n* = 267; Grades 8–10	Measured BMI classified using Asia‐Pacific criteria; overweight and obesity reported separately	Overweight: 14.6%; obesity: 14.6%; combined: 29.2%	Female sex: AOR 5.2 (95% CI 2.5–10.9); physical inactivity was also associated	Small rural school sample; use of adult‐type Asia‐Pacific BMI criteria limits comparability with age‐ and sex‐specific adolescent references
Sultana et al. (2021) [22]	Twenty‐country Global School‐based Student Health Survey, including Bangladesh and Nepal; cross‐sectional study	*n* = 51,597; 13–17 years	BMI‐based combined overweight/obesity; self‐reported height and weight	18.2% in the complete multicountry sample	Physical inactivity: OR 1.19; sedentary behaviour > 1 h/day: OR 1.25	Large school‐based analysis; however, the prevalence represents the entire 20‐country sample and should not be interpreted as a South Asia‐specific estimate unless country‐specific data are extracted
Karuppiah (2018) [23]	Sri Lanka; national schools in Batticaloa District; cross‐sectional study; girls only	*n* = 310; 14–19 years	BMI‐defined overweight and obesity; waist‐based central obesity	Overweight: 9.4%; obesity: 5.5%; central obesity: 21.6%	Family history of NCDs was reported among 70.6% of participants with obesity; central obesity was also identified among some participants with normal BMI	Local girls‐only sample; the family‐history finding is descriptive and should not be interpreted as an adjusted association
Jayarajan and Abirami (2020) [24]	India; Korkadu, Puducherry; small descriptive case–control study described by the authors as retrospective	*n* = 60: 30 participants with obesity and 30 controls; mean age 18.9 ± 2.3 years in cases and 18.06 ± 1.5 years in controls; complete age range not reported	Self‐reported height and weight; participants classified using adult WHO BMI thresholds; biochemical markers compared between obesity and control groups	Not applicable because participants were selected according to obesity status	Sedentary lifestyle and junk‐food consumption were identified by the authors as potential associated factors, but no numerical comparisons, effect estimates, CIs or *p*‐values were reported for these exposures. Family history was collected but its results were not reported. Glucose, total cholesterol and LDL cholesterol were significantly higher in the obesity group	Retained because the article explicitly described the participants as adolescents and the reported mean ages were within the WHO adolescent age range. Nevertheless, the complete age range was unavailable, adult BMI thresholds were used, anthropometry was self‐reported, and behavioural associations were not supported by adequately reported statistical analyses. The behavioural findings were therefore treated as suggestive rather than confirmed
Fahim et al. (2023) [25]	India; high schools in Raichur City; cross‐sectional study	*n* = 1900; 13–18 years	Measured BMI; overweight and obesity reported separately	Overweight: 4.57%; obesity: 5.11%	Age, sex, socioeconomic position, parental education, dietary practices and physical activity were reported as associated with weight status	Large local school sample; adjusted estimates should be reported where available, and the direction of sex‐specific findings requires clarification
Ahmed et al. (2024) [26]	Bangladesh; nationally representative Bangladesh Adolescent Health and Wellbeing Survey; cross‐sectional study	*n* = 9128; 15–19 years	WHO 2007 BMI‐for‐age; combined overweight/obesity defined as > +1 SD	Combined: 10.61% (95% CI 9.79–11.49); girls: 11.63%; boys: 8.25%; urban: 15.66%; semi‐urban: 8.69%; rural: 9.21%	Grade ≥ 11: adjusted OR 1.67; media exposure: adjusted OR 1.39; urban residence and higher household wealth were also independently associated	Nationally representative and methodologically strong primary study; overweight and obesity were analysed as a combined outcome
Hossain et al. (2020) [20]	Bangladesh; selected schools in Dhaka; cross‐sectional study	*n* = 150; 9–14 years	Measured BMI; overweight and obesity reported separately	Overweight: 36.0%; obesity: 25.3%; combined: 61.3%	Sedentary behaviour ≥ 120 min/day, limited sports participation and preference for fast food were associated with excess weight	Small selected urban sample with an overlapping age range; retained as contextual primary evidence but not considered directly comparable with adolescent‐only or nationally representative estimates

Abbreviations: AOR, adjusted odds ratio; BMI, body mass index; CI, confidence interval; GSHS, Global School‐based Student Health Survey; LDL, low‐density lipoprotein; NCD, noncommunicable disease; OR, odds ratio; SD, standard deviation; WHO, World Health Organization.

Most primary studies were school‐based or conducted in geographically restricted populations. Only one study used nationally representative data from Bangladesh [26], while the multicountry analysis used nationally representative school‐based survey data [22]. The remaining studies were predominantly local or subnational. No eligible primary studies were identified from Afghanistan, Bhutan, the Maldives or Pakistan during the original search period, highlighting important geographical gaps in the available evidence.

Participant age ranges, sex distributions and study settings varied considerably. Most studies included adolescents within the 10–19‐year age range, although one Bangladesh study included participants aged 9–14 years [20]. Jayarajan and Abirami explicitly described their participants as adolescents and reported mean ages of 18.9 ± 2.3 years in the obesity group and 18.06 ± 1.5 years in the control group, but did not provide the complete age range [24]. Both studies were retained because their populations predominantly or substantially overlapped with the WHO adolescent age range; however, their findings were interpreted cautiously. The studies also differed in whether they examined girls only, boys and girls together, or sex‐stratified outcomes.

The definitions of excess adiposity were heterogeneous. Most studies used BMI or BMI‐for‐age criteria, but the applied reference systems included WHO, IOTF, CDC, Asia‐Pacific, national or adult BMI classifications. Jayarajan and Abirami used self‐reported height and weight and applied adult WHO BMI thresholds to a sample described as adolescents [24]. Its findings were therefore not considered directly comparable with those based on age‐ and sex‐specific BMI classifications. One Sri Lankan study additionally reported central obesity using waist circumference [23]. Overweight, obesity, combined overweight/obesity and central obesity were therefore treated as distinct outcomes wherever the original data permitted.

The five review‐level publications comprised three systematic reviews [6, 16, 17] and two systematic reviews with meta‐analysis [5, 7] (Table 2). These reviews covered broader age ranges and included evidence from multiple South Asian countries. Because some reviews included both children and adolescents and potentially contained primary studies also identified in the present review, review‐level findings were analysed separately and used for contextual comparison rather than being combined with the primary‐study findings.

**TABLE 2:** Characteristics of review‐level evidence on overweight and obesity in South Asian children and adolescents.

Review	Countries and evidence base	Population and outcomes	Principal findings	Contribution and limitations
Mistry and Puthussery (2015) [16]	Systematic review of 11 school‐based studies from India, Pakistan and Bangladesh	Children and adolescents aged 0–18 years; overweight and obesity defined using heterogeneous criteria	Physical inactivity, sedentary behaviour, frequent consumption of fast food or energy‐dense foods, higher socioeconomic position and family history of obesity were commonly reported as associated factors	Provides an early regional synthesis of associated factors. Generalisability is limited by school‐based, predominantly cross‐sectional evidence from only three South Asian countries and by heterogeneous adiposity definitions
Ahmad et al. (2018) [6]	Systematic review of 12 studies from South Asian countries	Children and adolescents; BMI‐defined overweight and obesity	The review linked increasing obesity with dietary transition, reduced physical activity, sedentary lifestyles, urbanisation and changing family and social environments	Broadens the interpretation of obesity beyond individual behaviour, but combines different age groups, countries and study designs, limiting adolescent‐specific comparisons
Biswas et al. (2017) [7]	Systematic review and meta‐analysis of 20 studies from Bangladesh; approximately 123,887 participants	Participants aged 0–19 years; overweight and obesity	Pooled prevalence was approximately 7.0% for overweight and 6.0% for obesity. Prevalence varied by age, setting, time period and classification system	Provides country‐level pooled estimates, but combines children and adolescents and includes heterogeneous populations, periods and anthropometric definitions
Bansal et al. (2024) [5]	Systematic review and meta‐analysis of 152 studies involving 489,525 participants from seven South Asian countries	Children and adolescents aged ≤ 19 years; overweight, obesity and combined overweight/obesity	Pooled prevalence estimates were 12.4% for overweight, 6.6% for obesity and 19.3% for combined overweight/obesity. Obesity prevalence increased with publication year, indicating an upward regional trend	Provides the most extensive recent regional prevalence synthesis. Interpretation remains limited by substantial heterogeneity, uneven country coverage, varying age ranges and multiple classification systems
Akbar et al. (2025) [17]	Systematic review of 11 studies from Pakistan; most studies were urban and school‐based	Children and adolescents aged 0–18 years; overweight and obesity	Reported prevalence ranged from 0.5% to 23.0% for overweight and from 5.0% to 15.2% for obesity. Associated factors included insufficient physical activity, inadequate outdoor play, prolonged screen time, energy‐dense diets and unmonitored eating practices	Provides recent Pakistan‐specific evidence, but estimates were derived mainly from local, cross‐sectional school samples and were not pooled because of methodological heterogeneity

*Note:* Systematic reviews and meta‐analyses were analysed separately from primary studies and used only to contextualise regional prevalence, associated factors, and evidence gaps. They were not treated as independent primary observations or included in the synthesis of associations, thereby minimising overlap and double‐counting of evidence.

Abbreviation: BMI, body mass index.

Methodological quality was evaluated using design‐appropriate Joanna Briggs Institute (JBI) critical appraisal checklists [28]. The JBI checklist for analytical cross‐sectional studies was applied to the seven cross‐sectional studies, while the JBI case–control checklist was applied to the case–control study (Tables S2A and S2B). Most cross‐sectional studies adequately described their populations, settings and adiposity outcomes; however, the identification and management of potential confounding factors and the reporting of non‐significant findings were inconsistent.

The small case–control study by Jayarajan and Abirami had substantial limitations relating to participant matching, behavioural‐exposure measurement, confounder control, self‐reported anthropometry and statistical analysis [24]. The study did not report quantitative comparisons for the sedentary‐lifestyle and junk‐food factors identified in its conclusion. These findings were therefore treated as suggestive rather than statistically confirmed. No study was excluded solely on the basis of methodological limitations; instead, the appraisal findings informed the weight given to each study during the narrative synthesis.

### 3.3 Prevalence of Overweight and Obesity Among Adolescents

Prevalence estimates varied substantially across the included studies (Figure 2). However, a single overall prevalence range was not considered appropriate because studies differed in age range, sex distribution, sampling frame, geographical setting, anthropometric measure, and classification system.

**FIGURE 2:**
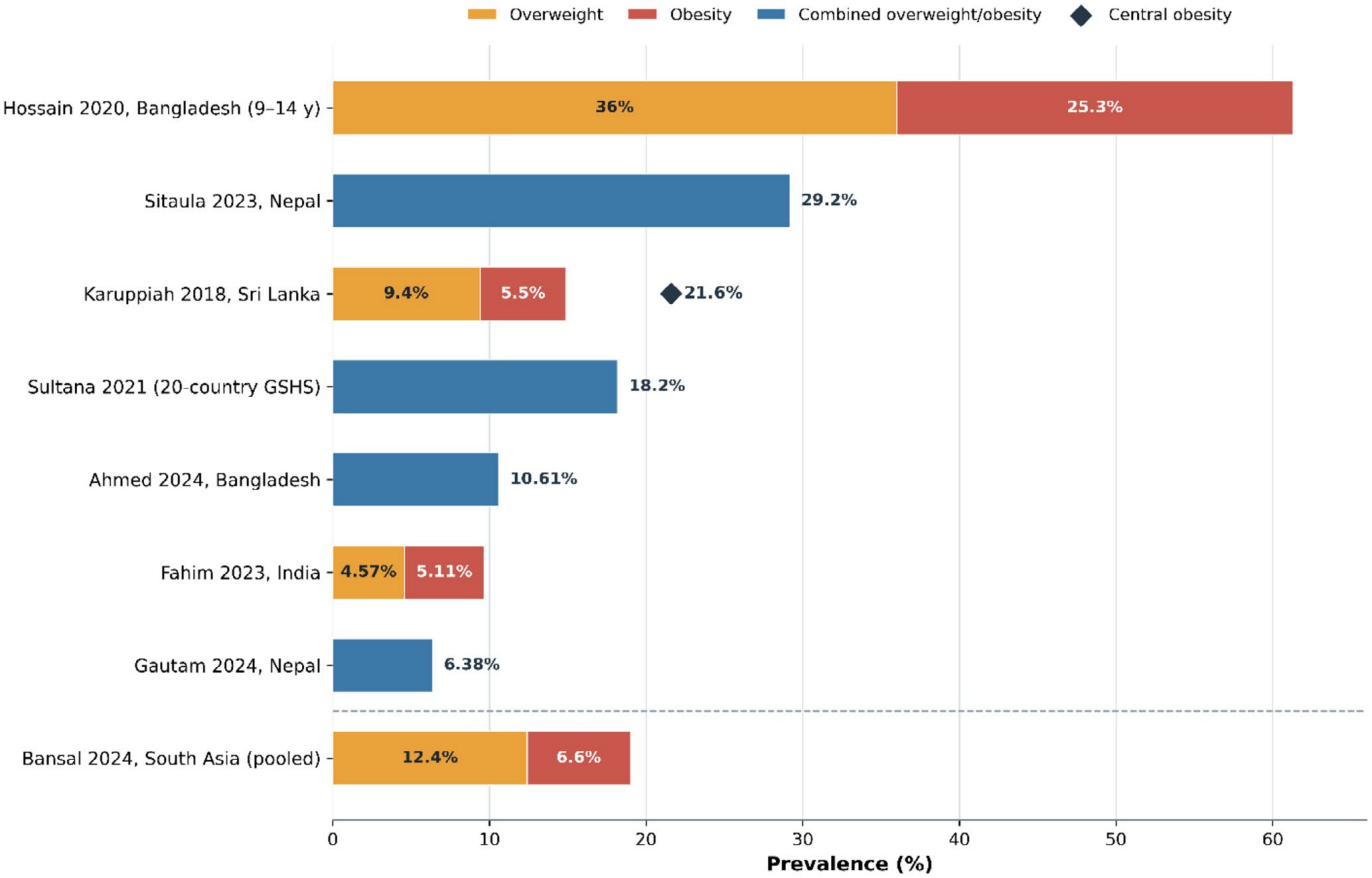
Prevalence of overweight, obesity, combined overweight/obesity and central obesity reported in selected South Asian studies. Estimates are presented according to the outcomes reported by each study. Hossain et al. included participants aged 9–14 years and was interpreted as contextual mixed‐age evidence. Sultana et al. reported findings from a 20‐country GSHS analysis that included Bangladesh and Nepal. The Bansal et al. estimate represents pooled review‐level evidence for South Asia. Differences in age, setting, sampling frame and adiposity classification limit direct comparison across studies.

Among the Nepalese studies, the prevalence of combined overweight/obesity ranged from approximately 6.4% in an urban municipality study to 29.2% in a smaller rural school‐based study [21, 27]. The wide difference between these estimates should be interpreted cautiously because the studies differed in sample size, population selection, and study setting.

In India, one school‐based study reported overweight and obesity prevalences of 4.57% and 5.11%, respectively [25]. Jayarajan and Abirami selected 30 participants with obesity and 30 controls and were therefore unsuitable for estimating population prevalence [24]. The study used self‐reported anthropometry and adult BMI thresholds, further limiting comparability with studies using BMI‐for‐age classifications.

In Sri Lankan adolescent girls, the reported prevalence was 9.4% for overweight, 5.5% for obesity and 21.6% for central obesity [23]. The higher estimate for central obesity illustrates that waist‐based measures and BMI‐based classifications identify different dimensions of adiposity and should not be directly combined.

Nationally representative data from Bangladesh indicated a combined overweight/obesity prevalence of 10.61% among adolescents aged 15–19 years [26]. The prevalence was higher among girls than boys (11.63% vs. 8.25%) and in urban than rural adolescents (15.66% vs. 9.21%), although these differences require consideration of socioeconomic and other contextual factors. By contrast, the mixed‐age Dhaka study reported overweight and obesity prevalences of 36.0% and 25.3%, respectively [20]. This estimate came from a small, selected urban sample aged 9–14 years and should not be interpreted as representative of Bangladeshi adolescents nationally.

At the review level, a recent South Asian meta‐analysis estimated pooled prevalences of 12.4% for overweight and 6.6% for obesity among children and young people [5]. The corresponding combined prevalence was approximately 19.3%. These pooled estimates included broader age ranges and multiple classification systems and therefore provide regional context rather than directly comparable adolescent‐specific estimates.

Overall, variation in prevalence appeared to reflect not only geographical differences but also differences in age, sex, socioeconomic position, urbanicity, sampling design, study period, anthropometric measurement, and diagnostic criteria. The evidence nevertheless indicates that adolescent overweight and obesity are important and increasingly visible public health concerns across South Asia.

### 3.4 Factors Associated With Overweight and Obesity

Factors identified across the eight primary studies were organised into domains covering physical activity, sedentary behaviour, dietary behaviours, sleep and psychosocial factors, familial and parental factors, sex and gender, socioeconomic position, rural–urban and built‐environment characteristics, and media or commercial influences. The direction and coverage of the reported associations are presented in Figure 3 and Table S3. Evidence was interpreted according to the magnitude and precision of reported associations, adjustment for confounding, sample size, representativeness and methodological quality, rather than by counting statistically significant findings.

**FIGURE 3:**
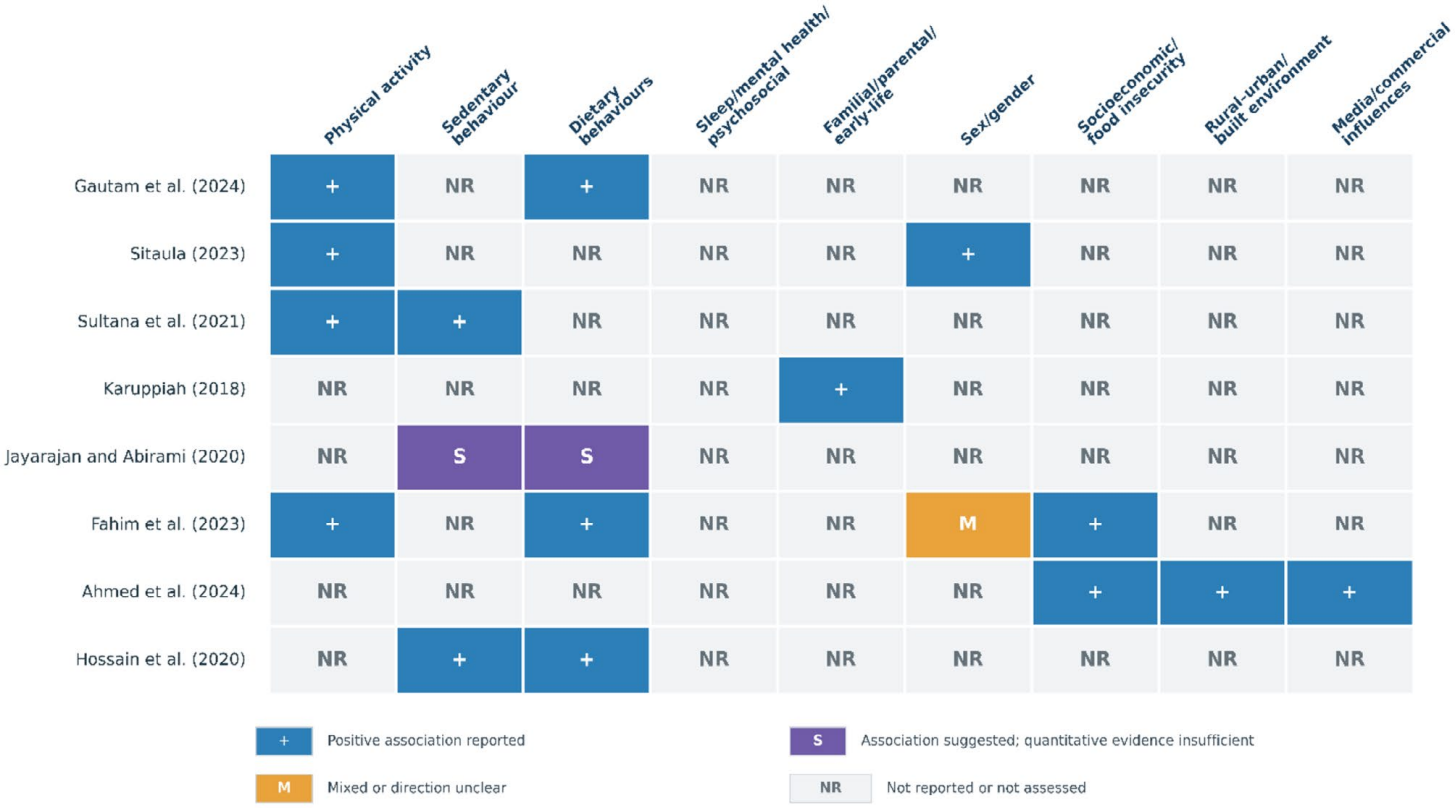
Evidence‐direction matrix of factors associated with adolescent overweight and obesity across the eight primary studies. Blue cells (+) indicate a positive association supported by the reported analysis; purple cells (S) indicate an association suggested by the authors but supported by insufficient quantitative evidence; orange cells (M) indicate mixed findings or an unclear direction; and grey cells (NR) indicate that the factor was not reported or apparently not assessed. NR should not be interpreted as evidence of no association. The matrix presents the direction and coverage of reported evidence rather than pooled effect sizes, comparative evidence strength or causal relationships.

#### 3.4.1 Physical Activity and Sedentary Behaviour

Evidence linking insufficient physical activity with adolescent overweight and obesity was broadly consistent, although exposure definitions varied across studies. Gautam et al. reported associations with lack of physical activity and absence of participation in school games [27]. Sitaula identified physical inactivity as an associated factor among rural school adolescents in Nepal [21]. In the large multicountry analysis by Sultana et al., physical inactivity was associated with increased odds of overweight or obesity (OR = 1.19), while sedentary behaviour exceeding 1 h per day was also associated with increased odds (OR = 1.25) [22]. Fahim et al. similarly reported an association with physical activity among Indian schoolchildren [25].

Sedentary behaviour was additionally examined in smaller, context‐specific studies. Jayarajan and Abirami identified sedentary lifestyle as a potential factor among adolescents with obesity in India; however, no study‐specific effect estimate or statistical comparison was reported for this exposure, and the finding was therefore considered suggestive [24]. Hossain et al. reported associations between excess weight and sedentary behaviour of at least 120 min per day, together with limited participation in sports, among schoolchildren in Dhaka [20]. Differences in exposure definitions, measurement methods, age ranges, and adjustment for potential confounding limited direct comparison across studies.

#### 3.4.2 Dietary Behaviours and Food Environments

Dietary evidence generally indicated associations between overweight or obesity and the consumption of energy‐dense or unhealthy foods, although dietary exposures were measured inconsistently. Gautam et al. identified consumption of high‐calorie lunches as an associated factor [27]. Jayarajan and Abirami identified junk‐food consumption as a potential factor among adolescents with obesity; however, no study‐specific effect estimate, confidence interval or statistical comparison was reported, and the finding was therefore considered suggestive [24]. Fahim et al. reported dietary practices as relevant correlates of overweight and obesity [25], while Hossain et al. found an association between excess weight and preference for fast food among schoolchildren in Dhaka [20].

Most studies assessed selected foods, food preferences or broadly defined dietary practices rather than validated dietary patterns, habitual energy intake or characteristics of the wider food environment. Differences in dietary assessment methods, exposure definitions and adjustment for potential confounding limited comparison across studies. Consequently, the available evidence suggests an association between unhealthy dietary behaviour and adolescent overweight or obesity but does not support firm conclusions about particular foods, consumption quantities, independent effects or causal dietary pathways.

#### 3.4.3 Sleep, Mental Health and Psychosocial Factors

Adolescent‐specific evidence concerning sleep duration and quality, mental health, stress, academic pressure and other psychosocial factors was insufficient. These exposures were either not assessed or not clearly reported in the included primary studies. This represents an important gap, particularly because these factors may interact with physical activity, sedentary behaviour and eating patterns.

#### 3.4.4 Familial, Parental and Early‐Life Factors

Karuppiah reported that 70.6% of adolescent girls with obesity had a family history of NCDs [23]. However, the small, local sample and absence of a clearly adjusted comparative estimate limit interpretation. Evidence concerning parental BMI, maternal health, infant feeding, birth weight and other early‐life factors was otherwise limited or not reported. Therefore, the contribution of familial and early‐life factors remains suggestive but insufficiently characterised.

#### 3.4.5 Sex‐ and Gender‐Related Factors

Sitaula reported substantially higher odds of adolescent obesity among girls than boys in a rural Nepalese sample (AOR = 5.2) [21]. Fahim et al. also reported an association with sex, although its direction and consistency across analyses were unclear [25]. Overall, sex‐related findings were mixed and appeared to vary by setting. The reviewed studies rarely distinguished biological sex from gender‐related influences such as mobility restrictions, opportunities for physical activity, household roles, or sociocultural expectations.

#### 3.4.6 Socioeconomic Position and Food Insecurity

Fahim et al. identified socioeconomic characteristics and parental education as factors associated with overweight and obesity [25]. In the nationally representative Bangladeshi study, Ahmed et al. reported a socioeconomic gradient, with a greater burden among adolescents from wealthier households [26]. Greater interpretive weight was assigned to the latter study because of its larger, nationally representative sample.

No included primary study provided sufficiently detailed evidence on household food insecurity or its possible coexistence with overweight and obesity. The available evidence therefore suggests an association with socioeconomic position but is insufficient to explain how household resources, food access and nutrition transition interact across different South Asian settings.

#### 3.4.7 Rural–Urban, Neighbourhood and Built‐Environment Factors

Ahmed et al. identified urban residence as an associated factor in Bangladesh [26]. Although prevalence was frequently higher in urban study populations, much of the evidence was derived from local school‐based samples, and few studies directly compared rural, peri‐urban and urban adolescents using consistent methods. Neighbourhood walkability, access to recreational spaces, transport patterns, food availability and other built‐environment characteristics were largely unexamined.

#### 3.4.8 Media Exposure and Commercial Influences

In the nationally representative Bangladeshi study, media exposure was associated with increased odds of overweight or obesity (OR = 1.39) [26]. However, evidence concerning exposure to food advertising, social media, digital marketing and other commercial influences was otherwise limited. Media exposure may also operate through increased sedentary time and exposure to unhealthy food promotion, but these pathways could not be evaluated using the available studies.

### 3.5 Summary of the Evidence

The comparatively more consistent evidence concerned insufficient physical activity, sedentary behaviour and unhealthy dietary practices, although the strength of support varied substantially across studies. However, heterogeneity in exposure definitions, adiposity classifications, study populations and confounder adjustment precluded direct quantitative comparison. Evidence concerning familial factors, sex and gender, socioeconomic position, urban residence and media exposure was more limited and context dependent.

Large or nationally representative studies provided more robust evidence for associations involving physical inactivity, sedentary behaviour, socioeconomic position, urban residence and media exposure, whereas findings from small or selected samples were interpreted cautiously. Important evidence gaps were identified for sleep, mental health, psychosocial stress, food insecurity, early‐life factors, neighbourhood environments and commercial determinants. Because most included studies were cross‐sectional, the reported associations should not be interpreted as causal relationships.

## 4 Discussion

This narrative review synthesised recent evidence on the prevalence of and factors associated with overweight and obesity among adolescents in South Asia. The findings indicate substantial variation in prevalence according to country, age, sex, sampling frame, residential setting, anthropometric measure and classification system. Physical inactivity, sedentary behaviour and unhealthy dietary practices showed comparatively consistent associations across multiple studies, although the strength and quality of support varied. More limited and context‐dependent evidence concerned socioeconomic position, urban residence, sex and gender, family history and media exposure.

These findings should not be interpreted causally. Most primary studies were cross‐sectional, school‐based and geographically restricted, and exposure definitions differed considerably. The available evidence therefore identifies associations and plausible pathways rather than definitive determinants of adolescent overweight or obesity.

### 4.1 Behavioural and Dietary Factors

Physical inactivity and sedentary behaviour were among the most frequently examined factors. Studies from Nepal, India, and Bangladesh reported associations between excess weight and limited physical activity, low participation in sports, prolonged sitting or screen‐based recreation [20, 21, 22, 25, 27]. Jayarajan and Abirami additionally identified a sedentary lifestyle as a potential factor among adolescents with obesity, but did not provide a study‐specific quantitative analysis of this association; their finding was therefore considered suggestive [24]. These findings are broadly consistent with evidence from other regions, where reduced moderate‐to‐vigorous physical activity and increased recreational screen time have been associated with excess adiposity among adolescents [29, 30, 31].

However, physical inactivity and sedentary behaviour are related but distinct exposures. An adolescent may meet recommended physical‐activity levels while also spending prolonged periods sitting. Conversely, low screen time does not necessarily indicate adequate physical activity. Several included studies did not distinguish these behaviours clearly, and the thresholds used to define inactivity or excessive screen time varied.

Academic expectations may be particularly relevant in South Asia. Long school hours, private tutoring, examination preparation and limited time for recreation may increase sedentary time. Opportunities for physical activity may also be constrained by inadequate school facilities, unsafe neighbourhoods, traffic, limited public spaces and gender‐related restrictions on outdoor mobility. Nevertheless, these mechanisms were rarely measured directly and should be considered plausible contextual explanations rather than established causal pathways.

Unhealthy dietary practices—including frequent fast‐food consumption, energy‐dense snacks, fried foods and high‐calorie school meals—were associated with overweight or obesity in several studies [20, 24, 25, 27]. The available primary evidence was less informative regarding portion size, total energy intake, meal timing, sugar‐sweetened beverages and ultra‐processed foods because dietary exposures were measured inconsistently and commonly relied on brief self‐reported questions.

### 4.2 Nutrition Transition and Commercial Food Environments

The dietary findings should be interpreted within South Asia's rapid and uneven nutrition transition. Traditional diets are increasingly accompanied or displaced by packaged snacks, fast foods, confectionery products and sugar‐sweetened beverages that are energy‐dense but often nutritionally poor. Urbanisation, rising household purchasing power, expansion of supermarkets and convenience stores, online food‐delivery services and changing family routines may increase adolescents' access to these products [11, 12].

Commercial influences are also important. Adolescents are exposed to food marketing through television, social media, online entertainment, sports sponsorship and digital platforms. Such marketing may influence brand preferences, purchasing requests and perceptions of normal eating behaviour. The observed association between media exposure and excess weight in Bangladesh may reflect sedentary time, exposure to food advertising, socioeconomic position or a combination of these pathways [26]. The available studies did not adequately distinguish these mechanisms.

These findings suggest that nutrition education alone is unlikely to be sufficient. Potential structural responses include improving school food environments, restricting the marketing of unhealthy foods to children and adolescents, promoting clear nutrition labelling, limiting the availability of sugar‐sweetened beverages in schools, and improving access to affordable, culturally appropriate, and nutritious foods.

### 4.3 Socioeconomic and Rural–Urban Differences

Higher socioeconomic position was associated with overweight or obesity in several South Asian studies [16, 25, 26]. This pattern differs from many high‐income settings, where obesity increasingly affects socioeconomically disadvantaged populations. In several South Asian settings, higher household wealth may still facilitate greater access to energy‐dense commercial foods, motorised transport, screen‐based entertainment and sedentary lifestyles, reflecting an earlier or intermediate stage of nutrition transition.

The socioeconomic gradient is unlikely to remain static. As inexpensive ultra‐processed foods become more widely available, lower‐income households may simultaneously experience food insecurity, inadequate dietary diversity and excess consumption of energy‐dense products. Future studies should therefore examine socioeconomic position using multiple indicators—including household wealth, income, parental education, occupation and food security—rather than treating it as a single uniform exposure.

Urban residence was associated with higher prevalence or odds of excess weight in several sources [5, 16, 26]. Urban residence should be viewed as a proxy for multiple food, transport, occupational and built‐environment exposures rather than as an independent causal factor. Adolescents living in urban settings may have greater access to fast food and motorised transport but fewer safe opportunities for outdoor activity. However, low‐income urban communities may also experience overcrowding, unsafe neighbourhoods, food insecurity and limited access to healthy foods.

The rural–urban distinction is also becoming less clear as packaged foods, smartphones, internet access and motorised transport expand into rural and peri‐urban communities. Future research should directly measure walkability, traffic, neighbourhood safety, recreational facilities, school environments and proximity to food outlets.

### 4.4 Familial, Biological, Sex‐ and Gender‐Related Factors

Evidence concerning familial and parental factors was limited. Karuppiah reported that 70.6% of adolescent girls with obesity had a family history of NCDs, but this was a descriptive finding without a comparative or adjusted estimate [23]. Jayarajan and Abirami collected family‐history information but did not report the corresponding results; their study therefore did not provide evidence of an association with family history or parental obesity [24]. Review‐level evidence nevertheless suggests that parental obesity and family history may be relevant in some South Asian populations [16]. These relationships may reflect genetic susceptibility, shared dietary and physical‐activity practices, cultural perceptions of body size and household socioeconomic conditions, but their independent contributions could not be determined from the available primary studies.

Sex‐related findings were inconsistent. One Nepalese study reported markedly higher adjusted odds among girls [21], while other studies reported smaller differences, no association, or higher prevalence among boys. Female sex should therefore not be characterised as a universal risk factor. Differences may reflect puberty, body composition, sociocultural norms, household responsibilities, safety concerns, and unequal opportunities for sport or outdoor recreation. In some settings, boys may instead have greater exposure to food purchased outside the home, sugar‐sweetened beverages, and recreational screen use.

Few studies formally tested whether associations between behavioural or socioeconomic factors differed by sex. Future research should report sex‐disaggregated estimates and directly measure gender‐related conditions rather than including sex only as an adjustment variable.

### 4.5 Double Burden of Malnutrition

Adolescent overweight and obesity in South Asia occur alongside persistent thinness, stunting, and micronutrient deficiencies. These forms of malnutrition may coexist across communities and households and, in some cases, within the same individual. An adolescent with overweight may still have iron, vitamin, or other micronutrient deficiencies because excess energy intake does not necessarily indicate adequate diet quality.

The double burden also has a life‐course dimension. Early‐life undernutrition followed by rapid weight gain and exposure to energy‐dense diets may contribute to central adiposity and later metabolic vulnerability. However, the included studies provided limited longitudinal evidence with which to evaluate these pathways directly.

Obesity‐prevention strategies in South Asia must therefore avoid simplistic approaches based solely on restricting energy intake. Policies should promote healthy growth, dietary quality, physical activity and metabolic health while protecting adolescents from undernutrition and micronutrient deficiency. School nutrition programmes, social‐protection initiatives and obesity‐prevention policies should be coordinated so that addressing one form of malnutrition does not inadvertently worsen another.

### 4.6 Comparison With Other Regions

Physical inactivity, sedentary behaviour and unhealthy dietary practices are associated with adolescent overweight and obesity worldwide and are not unique to South Asia. However, their distribution and expression may differ across social and economic contexts. Global evidence indicates substantial variation by country, sex, urbanicity, socioeconomic position, anthropometric measurement and classification system.

Recent pooled evidence suggests that the South Asian burden is substantial but numerically below corresponding global estimates. The South Asian meta‐analysis estimated prevalences of 12.4% for overweight, 6.6% for obesity and 19.3% for combined overweight/obesity among people aged 19 years or younger [5]. By comparison, a global systematic review of 2033 studies from 154 countries or regions, involving approximately 45.9 million participants younger than 18 years, estimated prevalences of 14.8% for overweight, 8.5% for obesity and 22.2% for combined excess weight [2]. However, these estimates should not be interpreted as demonstrating a statistically lower burden in South Asia because the two reviews differed in study periods, country composition, participant ages, sampling designs and adiposity classifications.

In many European and North American populations, excess weight is increasingly concentrated among socioeconomically disadvantaged groups, whereas higher socioeconomic position remains positively associated with obesity in several South Asian studies. Many African studies report higher prevalence among urban adolescents and, in some settings, among girls. East Asian and Pacific populations have experienced rapid increases associated with urbanisation, dietary transition and declining physical activity. These comparisons suggest that the socioeconomic and sex‐related patterning of adolescent obesity changes across stages of nutrition transition.

Cross‐regional comparisons nevertheless require caution. Global studies use different age groups, outcome definitions, BMI references, sampling frames and measurement methods. Some large school‐based surveys rely on self‐reported height and weight, whereas most South Asian primary studies used measured anthropometry. Moreover, overweight, obesity and combined overweight/obesity are not consistently reported as separate outcomes. Apparent regional differences may therefore partly reflect methodological heterogeneity rather than genuine biological, behavioural or environmental variation.

### 4.7 Emerging and Understudied Factors

Sleep duration, sleep quality, mental health, psychosocial stress, academic pressure, food insecurity and neighbourhood environments were insufficiently examined in the included South Asian studies. Short sleep may influence appetite, fatigue, meal timing and physical activity, while psychological distress may be associated with emotional eating, sedentary behaviour or disrupted sleep. Intensive academic schedules may further increase sitting time and reduce opportunities for recreation.

Food insecurity may have a complex relationship with obesity. Households with limited resources may have inadequate access to diverse, nutritious foods while relying on inexpensive energy‐dense products. Similarly, unsafe neighbourhoods and limited recreational spaces may restrict physical activity independently of individual motivation.

The limited evidence concerning these domains should not be interpreted as demonstrating that they are unimportant. Rather, it identifies a substantial gap in South Asian adolescent obesity research.

### 4.8 Implications for Policy and Practice

The findings support multilevel prevention strategies rather than interventions focused solely on individual behaviour. Schools remain an important setting for promoting physical activity and healthy eating, but school‐based approaches will not reach adolescents who have left school or attend irregularly. Community‐ and population‐based strategies are therefore also required.

Potential priorities include:

- Regular, inclusive and adequately resourced physical education.
- Safe spaces for recreation and active transport.
- Healthier school meals and restrictions on unhealthy foods within and around schools.
- Regulation of food marketing directed towards children and adolescents.
- Family‐centred nutrition and physical‐activity programmes.
- Integration of obesity prevention with programmes addressing undernutrition and mental health.
- Surveillance systems using standardised, measured anthropometry and sex‐disaggregated reporting.

Interventions should be adapted to national and local contexts because the prevalence and associated factors are unlikely to be uniform across South Asia.

### 4.9 Strengths and Limitations

This review has several strengths. It used a structured literature search, predefined eligibility criteria, and independent screening and appraisal. It also analysed primary studies separately from systematic reviews and meta‐analyses to reduce evidence overlap, distinguished among different adiposity outcomes, and considered methodological quality, representativeness, effect estimates, and precision rather than relying exclusively on statistical significance. Study‐level findings were classified according to whether an association was supported by a reported analysis, was only suggested by the study authors, or was mixed or insufficiently reported. The revised synthesis incorporates behavioural, dietary, psychosocial, familial, socioeconomic, sex‐ and gender‐related, environmental, and commercial perspectives.

Several limitations should be acknowledged. The review was restricted to English‐language publications and may therefore have missed relevant regional or non‐indexed literature. Google Scholar has limited reproducibility because its search results may vary over time and according to location and search history. The structured narrative approach was not based on a prospectively registered systematic‐review protocol, and the supplementary targeted search was intended to update and contextualise the evidence rather than recreate a complete systematic review. Consequently, this review should not be interpreted as providing an exhaustive systematic identification of all eligible regional studies.

Most primary studies were cross‐sectional, limiting temporal and causal inference. The evidence was concentrated in a small number of countries and was predominantly school‐based, potentially excluding adolescents who were not attending school. These adolescents may have different socioeconomic circumstances, occupational activity, marital or reproductive experiences, dietary exposures, and access to preventive services. Several studies used small, local, or highly selected samples, and only a limited proportion provided nationally representative evidence or adequately adjusted estimates.

Considerable heterogeneity existed in age ranges, sampling frames, anthropometric measures, classification criteria, exposure definitions, and analytical approaches. One mixed‐age study included participants aged 9–14 years and was retained as contextual primary evidence because most of its age range overlapped with adolescence; however, its findings were not interpreted as exclusively adolescent‐specific. Another small case–control study was retained because the authors explicitly described the participants as adolescents and the reported mean ages were within the WHO adolescent age range, although the complete age range was not provided. That study also used self‐reported anthropometry and adult BMI thresholds and did not report quantitative comparisons for the behavioural factors identified in its conclusion. Its dietary and sedentary‐behaviour findings were therefore treated as suggestive rather than statistically confirmed. Some review‐level estimates combined children and adolescents and could not be interpreted as adolescent‐specific prevalence. Because the included systematic reviews and meta‐analyses were used only for contextual comparison and were not formally appraised, prevalence estimates and broader conclusions derived from these sources should be interpreted cautiously.

Differences among WHO, IOTF, CDC, Asia‐Pacific, national and adult BMI classifications further limited comparison of prevalence estimates. In addition, overweight, obesity, combined overweight/obesity, and central obesity were not directly interchangeable and were therefore synthesised separately wherever possible. Dietary, physical‐activity, sedentary‐behaviour, media‐exposure, and socioeconomic measures were also inconsistently defined, restricting comparison of the magnitude of associations across studies.

Finally, the absence of published evidence from some countries or exposure domains should not be interpreted as the absence of an association. Publication bias, selective reporting of statistically significant findings, and inadequate reporting of null results may have influenced the available literature. Because many articles did not report all examined exposures, an unreported factor could not always be distinguished from a factor that was assessed but found to be non‐significant. Accordingly, cells classified as ‘not reported or not assessed’ in the evidence‐direction matrix should not be interpreted as evidence of no association.

### 4.10 Future Research Directions

Future research should prioritise:

- Prospective cohort studies capable of establishing temporal relationships.
- Nationally representative studies from Afghanistan, Bhutan, Maldives, and other underrepresented settings.
- Population‐based sampling that includes out‐of‐school adolescents.
- Standardised use and reporting of BMI‐for‐age classifications.
- Separate reporting of overweight, obesity and central obesity.
- Sex‐disaggregated analyses and assessment of gender‐related pathways.
- Validated measurement of diet, physical activity, sedentary behaviour and sleep.
- Assessment of mental health, academic pressure, food insecurity and neighbourhood environments.
- Evaluation of commercial food marketing and digital‐media exposure.
- Rigorous assessment of multilevel school, family, community and policy interventions.

Greater methodological standardisation would improve comparability, while country‐specific analyses would prevent regional averages from obscuring important local differences.

### 4.11 Conclusion

Adolescent overweight and obesity represent a growing public health and metabolic‐health challenge across South Asia. The comparatively more consistent evidence indicates associations with physical inactivity, sedentary behaviour and unhealthy dietary practices, while evidence concerning socioeconomic position, urban residence, sex and gender, familial characteristics and media exposure remains more limited and context dependent. However, the direction and strength of these associations vary across populations and settings.

The predominance of cross‐sectional, school‐based and geographically restricted studies prevents firm conclusions regarding causality or regional uniformity. Prevention strategies should therefore combine individual and family support with improvements in school, food and built environments while recognising the continuing double burden of malnutrition. Standardised longitudinal and population‐based research—including out‐of‐school adolescents and underrepresented countries—is essential for designing equitable and context‐specific responses across South Asia.

## Author Contributions

**Tasnima Siddiquee:** conceptualization, methodology, project administration, visualization, writing – original draft, data curation, formal analysis. **Bishwajit Bhowmik:** conceptualization, methodology, data curation, investigation, visualization, writing – original draft, project administration, formal analysis. **Nayla Cristina Do Vale Moreira:** writing – review and editing, conceptualization. **Sanjida Binte Munir:** data curation, visualization, writing – review and editing. **Hafiza Nasrin:** methodology, data curation, writing – review and editing. **Tanjimul Islam:** visualization, data curation, supervision, writing – review and editing. **Fahmida Mahmud:** data curation, software, visualization, resources. **Liya Quamrun Nesa:** data curation, visualization, resources. **Shamima Akhtar:** software, supervision, project administration. **Shamim Khan:** software, supervision, project administration. **Farsana Rahman Khan:** validation, supervision. **Mohammad Robed Amin:** writing – review and editing. **Abul Kalam Azad Khan:** conceptualization, writing – review and editing.

## Funding

The authors have nothing to report.

## Disclosure

The authors confirm that the manuscript does not reproduce copyrighted tables, figures or other material requiring third‐party permission. All figures were developed by the authors from the evidence synthesised in this review. A generative artificial intelligence tool was used to support language editing, structural organisation, and refinement of responses to peer‐review comments. All scientific content, interpretations, references, and revisions were critically reviewed and verified by the authors, who take full responsibility for the final manuscript.

## Ethics Statement

This review used data exclusively from previously published studies and did not involve the recruitment of human participants, collection of identifiable information, or use of animals. Therefore, institutional ethics approval and informed consent were not required.

## Conflicts of Interest

The authors declare no conflicts of interest.

## Supporting information


**Table S1:** Detailed literature search strategy.
**Table S2A:** JBI critical appraisal of analytical cross‐sectional studies.
**Table S2B:** JBI critical appraisal of the case–control study.
**Table S3:** Study‐level evidence on factors associated with adolescent overweight and obesity.

## Data Availability

No new participant‐level data were generated or analysed in this narrative review. All evidence was derived from previously published sources cited in the manuscript. The study characteristics and evidence extraction information supporting the narrative synthesis are provided in the main and supplementary tables. Additional details may be obtained from the corresponding author upon reasonable request.
