# Supplementary material for: Factors Associated With Overweight and Obesity Among Adolescents in South Asia: A Narrative Review of Recent Evidence
Source: Endocrinol Diabetes Metab. 2026 Oct 22;9(6):e70372. doi: 10.1002/edm2.70372 (PMC13690297; doi:10.1002/edm2.70372)
Supplement: Supplementary file 1 [file EDM2-9-e70372-s001.docx]

| **Number** | **Title** | **Page** |
| --- | --- | --- |
| Supplementary Table S1 | Detailed Literature Search Strategy | 2 |
| Supplementary Table S2A | JBI Critical Appraisal of Analytical Cross-Sectional Studies | 5 |
| Supplementary Table S2B | JBI Critical Appraisal of the Case-Control Study | 5 |
| Supplementary Table S3 | Study-Level Evidence on Factors Associated with Adolescent Overweight and Obesity | 6 |

| **Supplementary Table S1 \| Detailed Literature Search Strategy** | | |
| --- | --- | --- |
| **Database/source** | **Search period and update** | **Search strategy** |
| **PubMed** | Initial search: March 2025; supplementary sensitivity search: August 2026 | ("Obesity"[Mesh] OR "Overweight"[Mesh] OR "Body Mass Index"[Mesh] OR "Waist Circumference"[Mesh] OR overweight[Title/Abstract] OR obesity[Title/Abstract] OR adiposity[Title/Abstract] OR "body mass index"[Title/Abstract] OR BMI[Title/Abstract] OR "BMI-for-age"[Title/Abstract] OR "central obesity"[Title/Abstract] OR "abdominal obesity"[Title/Abstract] OR "waist circumference"[Title/Abstract] OR "waist-to-height ratio"[Title/Abstract]) AND ("Adolescent"[Mesh] OR adolescent*[Title/Abstract] OR teenager*[Title/Abstract] OR youth[Title/Abstract] OR "young people"[Title/Abstract] OR "school-going adolescent*"[Title/Abstract]) AND ("Exercise"[Mesh] OR "Sedentary Behavior"[Mesh] OR "Diet"[Mesh] OR "Feeding Behavior"[Mesh] OR "Mental Health"[Mesh] OR "Stress, Psychological"[Mesh] OR "Food Insecurity"[Mesh] OR "Socioeconomic Factors"[Mesh] OR determinant*[Title/Abstract] OR "risk factor*"[Title/Abstract] OR predictor*[Title/Abstract] OR "associated factor*"[Title/Abstract] OR correlate*[Title/Abstract] OR "physical activity"[Title/Abstract] OR "physical inactivity"[Title/Abstract] OR "sedentary behaviour"[Title/Abstract] OR "sedentary behavior"[Title/Abstract] OR "screen time"[Title/Abstract] OR diet*[Title/Abstract] OR "nutrition transition"[Title/Abstract] OR "fast food"[Title/Abstract] OR "ultra-processed food*"[Title/Abstract] OR "sugar-sweetened beverage*"[Title/Abstract] OR sleep[Title/Abstract] OR "mental health"[Title/Abstract] OR stress[Title/Abstract] OR "academic pressure"[Title/Abstract] OR "food insecurity"[Title/Abstract] OR "socioeconomic status"[Title/Abstract] OR family[Title/Abstract] OR parental[Title/Abstract] OR neighbourhood[Title/Abstract] OR neighborhood[Title/Abstract] OR "built environment"[Title/Abstract]) AND ("Asia, Southern"[Mesh] OR "South Asia"[Title/Abstract] OR Afghanistan[Title/Abstract] OR Bangladesh[Title/Abstract] OR Bhutan[Title/Abstract] OR India[Title/Abstract] OR Maldives[Title/Abstract] OR Nepal[Title/Abstract] OR Pakistan[Title/Abstract] OR "Sri Lanka"[Title/Abstract]) |
| **Scopus** | Initial search: March 2025; supplementary sensitivity search: August 2026 | TITLE-ABS-KEY (overweight OR obesity OR adiposity OR "body mass index" OR BMI OR "BMI-for-age" OR "central obesity" OR "abdominal obesity" OR "waist circumference" OR "waist-to-height ratio") AND TITLE-ABS-KEY (adolescent* OR teenager* OR youth OR "young people" OR "school-going adolescent*") AND TITLE-ABS-KEY (determinant* OR "risk factor*" OR predictor* OR "associated factor*" OR correlate* OR "physical activity" OR "physical inactivity" OR "sedentary behaviour" OR "sedentary behavior" OR "screen time" OR diet* OR "nutrition transition" OR "fast food" OR "ultra-processed food*" OR "sugar-sweetened beverage*" OR sleep OR "mental health" OR stress OR "academic pressure" OR "food insecurity" OR "socioeconomic status" OR family OR parental OR neighbourhood OR neighborhood OR "built environment") AND TITLE-ABS-KEY ("South Asia" OR Afghanistan OR Bangladesh OR Bhutan OR India OR Maldives OR Nepal OR Pakistan OR "Sri Lanka") |
| **Google Scholar** | Initial search: March 2025; supplementary targeted searches: August 2026 | Combinations of: ("adolescent obesity" OR "adolescent overweight" OR "central obesity" OR "abdominal obesity") AND ("associated factors" OR "risk factors" OR determinants OR predictors) AND ("South Asia" OR Afghanistan OR Bangladesh OR Bhutan OR India OR Maldives OR Nepal OR Pakistan OR "Sri Lanka"). Additional targeted searches combined individual country names with the following exposure domains: physical activity, physical inactivity, sedentary behaviour, screen time, diet, nutrition transition, fast food, ultra-processed food, sugar-sweetened beverages, sleep, mental health, stress, academic pressure, food insecurity, socioeconomic position, familial or parental factors, neighbourhood environment, built environment, media exposure and commercial food marketing. |
| **Reference-list screening** | Initial screening: March 2025; updated: August 2026 | Reference lists of eligible primary studies and relevant systematic reviews and meta-analyses were manually screened to identify additional potentially eligible publications. |
| **Contextual global and non-South Asian evidence** | Targeted update: August 2026 | Targeted searches combined adolescent overweight or obesity with prevalence, trends, physical activity, sedentary behaviour, dietary factors, nutrition transition, ultra-processed foods, sleep, mental health, socioeconomic position, food insecurity, urbanisation, neighbourhood and built environments, media exposure, and commercial influences. This evidence was used only for contextual comparison in the Introduction and Discussion and was not included in the primary South Asian synthesis. |
| Note: Search terms were adapted to the indexing conventions and syntax of each database. PubMed searches combined relevant Medical Subject Headings with free-text Title/Abstract terms. Searches were limited to peer-reviewed publications in English. The August 2026 supplementary sensitivity and targeted searches identified no additional eligible South Asian studies; therefore, the study-selection counts remained unchanged. Global and non-South Asian publications identified during the contextual search were not included in the primary synthesis or study-selection flow diagram. | | |

| **Supplementary Table S2A \| JBI Critical Appraisal of Analytical Cross-Sectional Studies** | | | | | | | | |
| --- | --- | --- | --- | --- | --- | --- | --- | --- |
| **Study** | **Q1** | **Q2** | **Q3** | **Q4** | **Q5** | **Q6** | **Q7** | **Q8** |
| Gautam et al. (2024) | Y | Y | Y | Y | Y | Y | Y | Y |
| Sitaula (2023) | Y | Y | Y | Y | Y | Y | Y | Y |
| Sultana et al. (2021) | Y | Y | Y | Y | Y | Y | Y | Y |
| Karuppiah (2018) | Y | Y | Y | Y | U | Y | Y | Y |
| Fahim et al. (2023) | Y | Y | Y | Y | Y | Y | Y | Y |
| Ahmed et al. (2024) | Y | Y | Y | Y | Y | Y | Y | Y |
| Hossain et al. (2020) | Y | Y | Y | Y | U | Y | Y | Y |
| JBI analytical cross-sectional checklist items: Q1, clearly defined inclusion criteria; Q2, study participants and setting described in detail; Q3, exposure measured validly and reliably; Q4, objective and standard criteria used to measure the condition; Q5, confounding factors identified; Q6, strategies to address confounding stated; Q7, outcomes measured validly and reliably; Q8, appropriate statistical analysis used**.**  Abbreviations: N, no; U, unclear; Y, yes. | | | | | | | | |

| **Supplementary Table S2B \| JBI Critical Appraisal of the Case-Control Study** | | | | | | | | | | |
| --- | --- | --- | --- | --- | --- | --- | --- | --- | --- | --- |
| **Study** | **Q1** | **Q2** | **Q3** | **Q4** | **Q5** | **Q6** | **Q7** | **Q8** | **Q9** | **Q10** |
| Jayarajan and Abirami (2020) | U | N | Y | N | U | N | N | N | U | N |
| JBI case-control checklist items: Q1, groups comparable except for the presence of the condition; Q2, cases and controls appropriately matched; Q3, the same criteria used to identify cases and controls; Q4, exposure measured in a standard, valid and reliable manner; Q5, exposure measured similarly for cases and controls; Q6, confounding factors identified; Q7, strategies to address confounding stated; Q8, outcomes assessed in a standard, valid and reliable manner; Q9, exposure period sufficiently meaningful; Q10, appropriate statistical analysis used.  Abbreviations: N, no; U, unclear; Y, yes. | | | | | | | | | | |

| **Supplementary Table S3 \| Study-Level Evidence on Factors Associated with Adolescent Overweight and Obesity** | | | | | |
| --- | --- | --- | --- | --- | --- |
| **Study** | **Evidence domain** | **Associated factor examined** | **Reported finding/effect estimate** | **Evidence direction** | **Interpretive considerations** |
| Gautam et al. (2024) [22] | Physical activity | Lack of physical activity | Reported as associated with overweight/obesity | Positive | Local cross-sectional study; temporality cannot be established |
| Gautam et al. (2024) [22] | Physical activity | Lack of participation in school games | Reported as associated with overweight/obesity | Positive | School-related activity measure; residual confounding possible |
| Gautam et al. (2024) [22] | Dietary behaviours | High-calorie lunch | Reported as associated with overweight/obesity | Positive | Dietary exposure was not based on comprehensive assessment of usual intake |
| Sitaula (2023) [23] | Physical activity | Physical inactivity | Reported as associated with adolescent obesity | Positive | Rural, school-based cross-sectional sample |
| Sitaula (2023) [23] | Sex and gender | Female sex | AOR = 5.2 | Positive | Large estimate from a relatively small, context-specific sample; precision should be interpreted using the reported 95% CI |
| Sultana et al. (2021) [24] | Physical activity | Physical inactivity | OR = 1.19 | Positive | Large multicountry school-based analysis; exposure and anthropometry were based on GSHS measures |
| Sultana et al. (2021) [24] | Sedentary behaviour | Sedentary behaviour >1 hour/day | OR = 1.25 | Positive | Large sample, but cross-sectional design and self-reported behaviours limit causal interpretation |
| Karuppiah (2018) [25] | Familial and parental factors | Family history of NCDs | Present among 70.6% of participants with obesity | Positive association suggested | Descriptive proportion rather than a clearly adjusted comparative effect estimate |
| Jayarajan and Abirami (2020) [26] | Sedentary behaviour | Sedentary lifestyle | Identified by the authors as a key associated factor, but no numerical comparison, CI or p-value was reported | Positive association suggested | Small case-control study; behavioral exposure measurement and confounder adjustment were insufficiently described |
| Jayarajan and Abirami (2020) [26] | Dietary behaviours | Junk-food consumption | Identified by the authors as a key associated factor, but no effect estimate, CI or p-value was reported | Positive association suggested | The magnitude and independence of the association cannot be determined |
| Jayarajan and Abirami (2020) [26] | Familial and parental factors | Family history | Collected by questionnaire, but results were not presented | NR | Measurement without reported results cannot be interpreted as evidence of association |
| Fahim et al. (2023) [27] | Physical activity | Physical activity level | Reported as associated with overweight/obesity | Positive | Cross-sectional school-based evidence |
| Fahim et al. (2023) [27] | Dietary behaviours | Dietary practices | Reported as associated with overweight/obesity | Positive | Dietary assessment and exposure definition require consideration |
| Fahim et al. (2023) [27] | Sex and gender | Sex | Association reported, but direction was not consistently clear across the extracted findings | Mixed/direction unclear | Sex-specific estimates should be reported where available |
| Fahim et al. (2023) [27] | Socioeconomic position | Socioeconomic status and parental education | Reported as associated with overweight/obesity | Positive/context dependent | Direction may differ across socioeconomic indicators |
| Ahmed et al. (2024) [28] | Socioeconomic position | Higher household wealth | Higher likelihood of overweight/obesity | Positive | Nationally representative study; comparatively strong contextual evidence |
| Ahmed et al. (2024) [28] | Rural–urban environment | Urban residence | Higher likelihood of overweight/obesity | Positive | Nationally representative data, although cross-sectional |
| Ahmed et al. (2024) [28] | Media and commercial influences | Media exposure | OR = 1.39 | Positive | Media exposure may capture both sedentary behaviour and exposure to marketing |
| Ahmed et al. (2024) [28] | Sociodemographic characteristics | Educational grade ≥11 | OR = 1.67 | Positive | May reflect age, educational exposure or correlated socioeconomic characteristics |
| Hossain et al. (2020) [29] | Sedentary behaviour | Sedentary behaviour ≥120 minutes/day | Reported as associated with overweight/obesity | Positive | Small, selected urban school sample |
| Hossain et al. (2020) [29] | Physical activity | Low participation in sports | Reported as associated with overweight/obesity | Positive | Potential overlap with sedentary-behaviour measures |
| Hossain et al. (2020) [29] | Dietary behaviours | Preference for fast food | Reported as associated with overweight/obesity | Positive | Food preference may not represent actual frequency or quantity consumed |
| **Abbreviations:** AOR, adjusted odds ratio; GSHS, Global School-based Student Health Survey; NCD, non-communicable disease; OR, odds ratio. **Note:** “Positive” indicates a positive association supported by a reported statistical analysis. “Association suggested” indicates that the factor was identified by the study authors, but adequate quantitative comparisons or effect estimates were not reported. “Mixed/direction unclear” indicates inconsistent findings or insufficient information to determine the direction of the association. Factors not reported or apparently not assessed are displayed as “NR” in Figure 3 but are not listed as evidence rows in this table. Neither “NR” nor absence from the table should be interpreted as evidence of no association. Effect estimates are presented with their corresponding 95% confidence intervals where available in the original articles. | | | | | |
